# Journey of the Nasal Septum Into the Lungs: A Unique Complication of Cocaine Abuse

**DOI:** 10.7759/cureus.9240

**Published:** 2020-07-17

**Authors:** Mohammed N Salem, Naureen Narula, Michel Chalhoub, Alaa Salem, Shimshon Wiesel

**Affiliations:** 1 Internal Medicine, Staten Island University Hospital, Staten Island, USA; 2 Internal Medicine, Staten Island University Hospital, Northwell Health, Staten Island, USA; 3 Medicine, Avalon University School of Medicine, Youngstown, USA; 4 Medicine, Staten Island University Hospital, Staten Island, USA

**Keywords:** cocaine ingestion, nasal septum perforation, drug abuse, cocaine abuse, auto-aspiration, aspiration pneumonia, nasal septum aspiration

## Abstract

Cocaine is a powerful addictive stimulant drug which is known to have a wide range of adverse effects across the spectrum of organ systems. Pulmonary complications have been widely reported. Intranasal inhalation has its own sequelae of complications. It is also quite common that patients who use cocaine also use other drugs. Polysubstance abuse may result in varying detrimental effects. Here, we report a unique case of nasal septal aspiration as a complication of intranasal cocaine inhalation with concomitant alcohol abuse. It is hypothesized the patient perforated his nasal septum due to chronic intranasal inhalation of cocaine and he subsequently aspirated his septum as a result of central nervous system depression secondary to alcohol intoxication.

## Introduction

Cocaine is a powerful addictive stimulant drug which is known to have a wide range of adverse effects across the spectrum of organ systems [1-2]. Pulmonary complications have been widely reported. Intranasal inhalation is associated with significant sequelae-sinusitis, epistaxis, and nasal septum necrosis being the most common [2-3]. Pulmonary effects depend on the route of administration (oral, nasal, intravenous) and concomitant abuse of other substances. Effects include airway injury, infectious and aspiration pneumonia, with the most common being productive cough of dark sputum (carbonaceous material), chest pain, dyspnea, hemoptysis, wheezing, and exacerbation of asthma [4]. Here, we report a case of nasal septal aspiration as a complication of intranasal cocaine inhalation with concurrent alcohol abuse. 

## Case presentation

**Figure 1:**
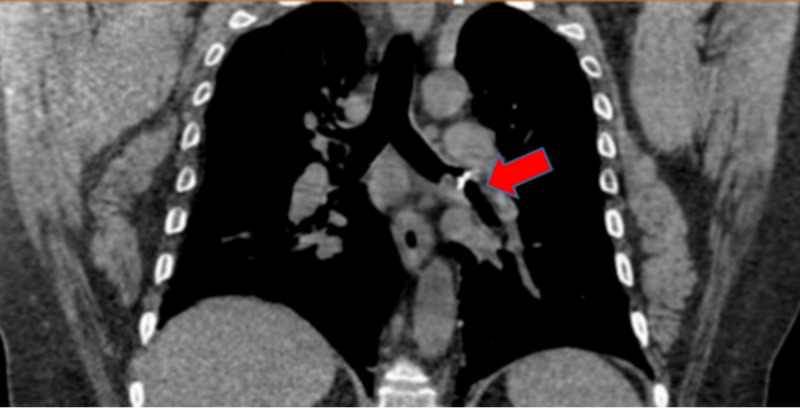
CT chest with 1 cm hyper-density (red arrow) at the bifurcation of the left main bronchus extending into the left upper lobe concerning for an aspirated FB, FB, foreign body

**Figure 2:**
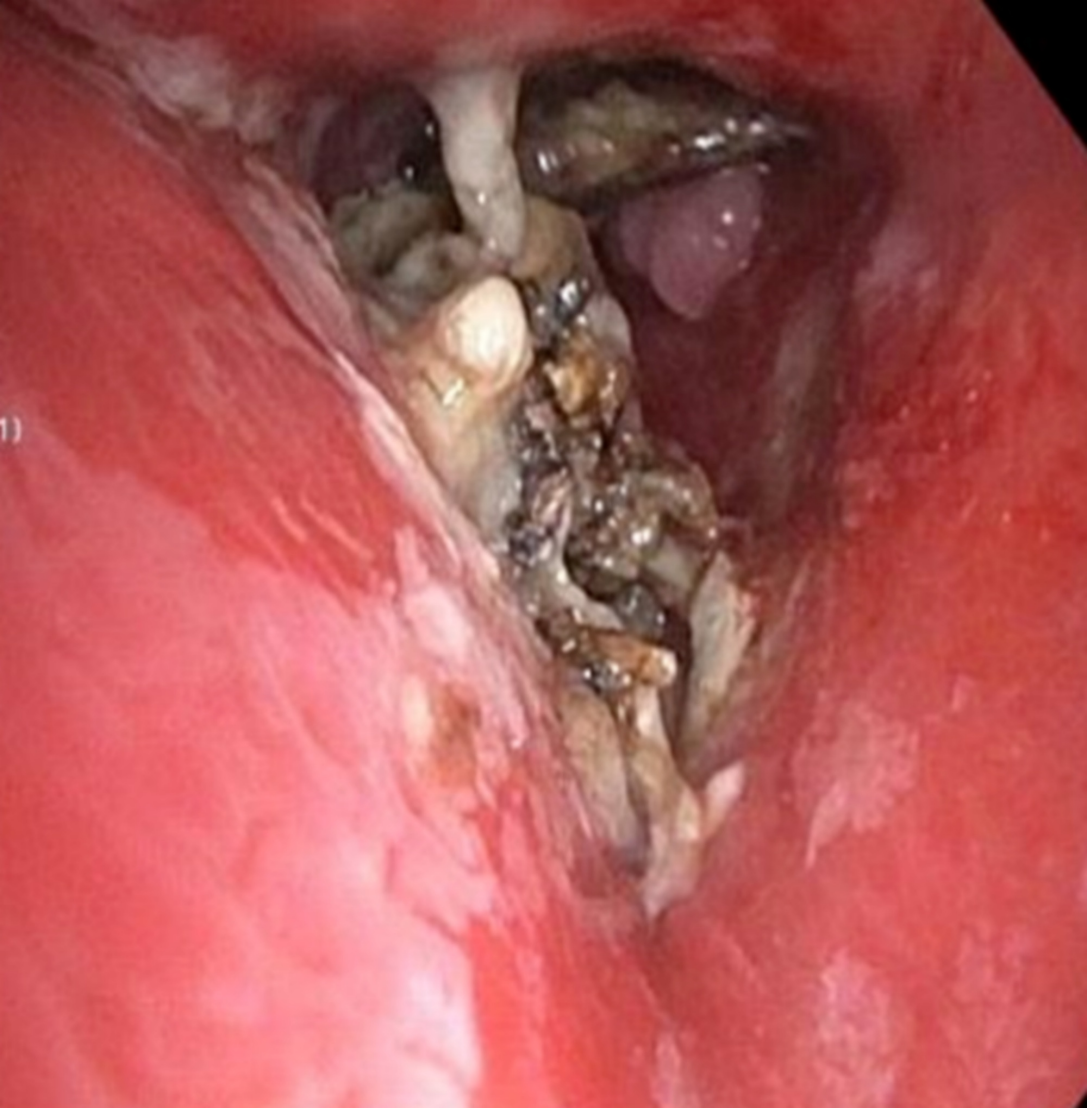
Image captured during bronchoscopy visualizing what appeared as necrotic mass.

A 48-year-old man, 30 pack year smoker with a history of chronic obstructive pulmonary disease (COPD) and cocaine abuse, presented with complaints of cough with minimal mucoid expectoration and wheezing associated with worsening shortness of breath for three months. Physical examination was within normal limits except for mild wheezing bilaterally, and lab work was unremarkable. Chest X-ray (CXR) was reported negative for acute cardiopulmonary pathology. Decision was made to obtain a CT chest which was consistent with 1 cm hyper-density noted at the bifurcation of the left main bronchus extending into the left upper lobe concerning for an aspirated foreign body (FB), this finding not noted on CXR (Figure 1). After this finding was discussed with the patient, he did recall recently breaking a tooth which led to consideration of FB (tooth) aspiration as the provisional diagnosis, but malignancy was not ruled out seeing his smoking history. Flexible bronchoscopy revealed a deeply embedded mass surrounded by significant vascular granulation tissue partially obstructing the left main bronchus, no tooth was visualized (Figure 2). Biopsy of the mass was performed and was reported as fragments of reactive squamous epithelium and nonviable appearing cartilage and bone consistent with nasal septal tissue. The patient’s nasal cavity was inspected by an otorhinolaryngologist and he was found to have a posterior nasal perforation so it was deduced that he had aspirated his own nasal septum.

 

## Discussion

Auto-aspiration of one's nasal septum is not a common complication seen. The history of intranasal inhalation of cocaine and concomitant alcohol use makes nasal septum necrosis and eventual dislocation of the septum into the airway unique to our patient. High index of suspicion by the clinician is required to order CT chest when CXR is negative due to a low diagnostic accuracy when suspecting aspiration of FB [5]. Rigid or flexible bronchoscopy should subsequently be performed to retrieve or biopsy the FB or mass respectively. As in our case, initial event can go unnoticed with persistent coughing being the most common symptom which can mimic COPD, asthma, or even obstructive pneumonia [4]. The patient did benefit from a course of glucocorticoids which is reported to reduce granulation tissue secondary to FB aspiration [6-7].

## Conclusions

To the best of our knowledge, this is second reported of nasal septum aspiration, the first being published in *Annals of Internal Medicine* in 1992. It is important for physicians to be aware about this entity specifically in patients using cocaine alone or with concurrent substances. High index of suspicion and adequate clinical history are important for achieving the correct diagnosis in this patient population. 
